# Polymeric Composite of Magnetite Iron Oxide Nanoparticles and Their Application in Biomedicine: A Review

**DOI:** 10.3390/polym14040752

**Published:** 2022-02-15

**Authors:** Moises Bustamante-Torres, David Romero-Fierro, Jocelyne Estrella-Nuñez, Belén Arcentales-Vera, Estefani Chichande-Proaño, Emilio Bucio

**Affiliations:** 1 Departamento de Biología, Escuela de Ciencias Biológicas e Ingeniería, Universidad de Investigación de Tecnología Experimental Yachay, Hacienda San José s/n y Proyecto Yachay (Ciudad del Conocimiento Yachay), Urcuqui 100650, Ecuador; 2 Departamento de Química de Radiaciones y Radioquímica, Instituto de Ciencias Nucleares, Universidad Nacional Autónoma de México, Circuito Exterior, Ciudad Universitaria, Mexico City 04510, Mexico; david.romero@yachaytech.edu.ec; 3 Departamento de Química, Escuela de Ciencias Química e Ingeniería, Universidad de Investigación de Tecnología Experimental Yachay, Hacienda San José s/n y Proyecto Yachay (Ciudad del Conocimiento Yachay), Urcuqui 100650, Ecuador; jocelyne.estrella@yachaytech.edu.ec (J.E.-N.); maria.arcentales@yachaytech.edu.ec (B.A.-V.); 4 Departamento Biología, Facultad de Biología, Universidad Central del Ecuador, Cuidad de Quito 170136, Ecuador; eachichande@uce.edu.ec

**Keywords:** iron oxide nanoparticles, superparamagnetic iron oxide nanoparticles, hydrogels, magnetic nanoparticles, nanocomposites, synthesis, characterization

## Abstract

A broad spectrum of nanomaterials has been investigated for multiple purposes in recent years. Some of these studied materials are magnetics nanoparticles (MNPs). Iron oxide nanoparticles (IONPs) and superparamagnetic iron oxide nanoparticles (SPIONs) are MNPs that have received extensive attention because of their physicochemical and magnetic properties and their ease of combination with organic or inorganic compounds. Furthermore, the arresting of these MNPs into a cross-linked matrix known as hydrogel has attracted significant interest in the biomedical field. Commonly, MNPs act as a reinforcing material for the polymer matrix. In the present review, several methods, such as co-precipitation, polyol, hydrothermal, microemulsion, and sol-gel methods, are reported to synthesize magnetite nanoparticles with controllable physical and chemical properties that suit the required application. Due to the potential of magnetite-based nanocomposites, specifically in hydrogels, processing methods, including physical blending, in situ precipitation, and grafting methods, are introduced. Moreover, the most common characterization techniques employed to study MNPs and magnetic gel are discussed.

## 1. Introduction

For a few decades, growing chemical synthesis of nanomaterials and material surface modification have been observed and performed in numerous applications, including biomedicine, biotechnology, catalysis, magnetic chemistry thermoelectric materials, etc. [1]. Nanoparticles (NPs) are a kind of nanomaterial that have attracted the interest of scientists. According to the synthesis method, NPs with different compositions, shapes, sizes, size distributions, and properties can be obtained. One of the most important and studied NPs is the MNP. The unique physicochemical properties of MNPs, especially their large surface areas, ease of synthesis and modification, and inherent superparamagnetic properties, could lead to improved technologies [2]. Moreover, these MNPs present an excellent capability to achieve a synergic union with other compounds, such as polymers [3].

Polymers can adopt a cross-linked matrix known as a hydrogel depending on the polymerization method. Hydrogels have widespread use in tissue engineering and functional devices because of their biomimetic properties and multi functionalities [4]. A hydrogel can be defined as a three-dimensional cross-linked polymer network that can absorb and retain a large amount of water [5] and active components [6], such as NPs.

Magnetic materials have been studied in recent years for their potent versatility. IONPs emerged as a promising material due to their magnetic properties, the superparamagnetism that leads to very high relativity, high biocompatibility, and easy functionalization of their surfaces with target molecules [7]. Several methods have been considered for synthetizing IONPs [8] by obtaining MNPs of different shapes and sizes. Moreover, based on their size, IONPs can be classified into three categories: micrometer-sized (300–3.5 µm), standard-sized (10–150 nm), and ultra-small (<10 nm) iron oxide crystals. Moreover, MNPs can be classified according to their magnetic properties as IONPs and SPIONs. Moreover, SPIONs are a kind of IONPs with remarkable magnetic properties. The great advantage of SPIONs is their magnetic properties that allow direct delivery of matter into the pathogen zone without influencing the whole organism [9].

The functionalization of the MNPs with organic and inorganic materials is indispensable for its applications. IONPs and SPIONs have an iron oxide core coated by an organic or inorganic layer [10] or encapsulated in a polymeric matrix. MNPs are usually functionalized with proteins (amino group), silica, polymer, surfactants, and organic materials to reduce toxicity and optimally fulfill their biomedical functions in drug delivery applications [11]. IONPs have been used to tailor the properties of polymeric hydrogels [12]; meanwhile, the hydrogels acquire a magnetic response. These hydrogels are subjected to reversible changes in their microstructure, as they can go from a compressed state to a swollen state by responding to an external magnetic field [13].

To implement MNCs in the biomedicine field, these must meet multiple requirements such as non-toxicity, biocompatibility, and stability [14]. The dispersity and stability of the IONPs in an aqueous medium is attained by coating their surface with hydrophilic polymers such as starch, and dextran [15]. Medical applications and biotechnological advances, including magnetic resonance imaging, cell separation and detection, tissue repair, magnetic hyperthermia and drug delivery, have strongly benefited from employing IONPs due to their remarkable properties, such as superparamagnetism, size, and the possibility of receiving a biocompatible coating [16]. Similarly, SPIONs are usually studied for targeting, imaging, biosensors, and drug delivery [17]

The synergic union between magnetic nanomaterials and polymers can form new materials known as composites. Moreover, the polymeric composites display exciting characteristics, which could be studied through several characterization techniques. Here, we review the function, structure, synthesis, characterization, and applications of a polymeric composite of magnetite IONPs.

## 2. Iron Oxide Nanoparticles (IONPs) and Superparamagnetic Iron Oxide Nanoparticles (SPIONs)

IONPs and SPIONs are attractive materials with excellent properties and magnetic tenability, making them suitable for biomedical approaches [18]. IONPs have attracted widespread attention due to their biocompatibility, low cost, chemically stable, and unique magnetic features. Meanwhile, SPIONs are IONPs with enhanced magnetic properties. In addition, SPIONs are nanomaterials characterized as chemically inert materials which decrease toxicity. They are characterized by a superparamagnetic character and immune induction capacity, making them a potential charger for antigen delivery [19].

**Figure 1:**
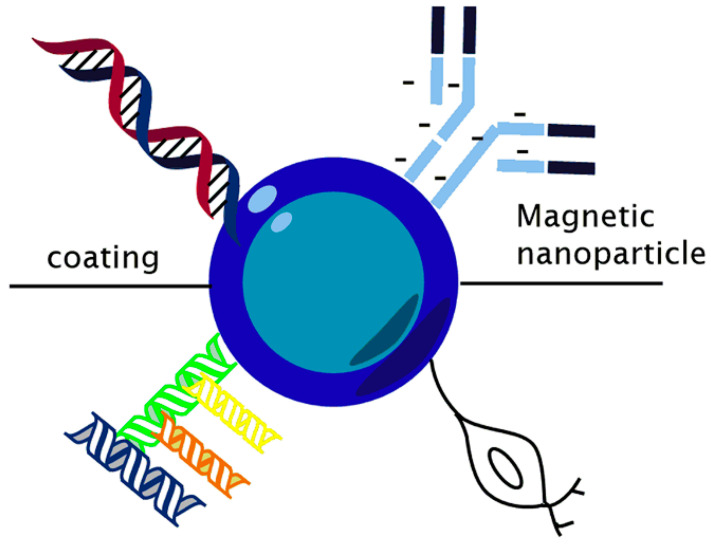
Schematic representation of magnetic nanoparticle.

These magnetic materials present unique intrinsic magnetic properties known as superparamagnetism and high colloidal stability, making them very attractive in a wide range of uses [20], with an approach to magnetic resonance imaging. Moreover, they can be obtained by employing different chemical methods, where IONPs without any surface coating (un-functionalized) are not stable in aqueous media, resulting in a readily aggregate and precipitate [21]. The colloidal suspension of iron oxides (un-functionalized), particularly magnetite, is easily oxidized in air and susceptible to loss of magnetism [11]. Similarly, bare SPIONs may be toxic because they are chemically reactive, so the coating layer prevents aggregation and agglomeration of the NPs and reduces iron oxide oxidation [10]. To overcome this issue, surface modification and a combination of other materials to develop new nanocomplexes through a highly engineered process can combat the physiological barrier [18]. For example, Figure 1 illustrates a general representation of MNPs combined with other materials.

The spinel ferrite has an MFe_2_O_4_ structure, where M is a divalent cation. This spinel ferrite has been recognized for its commercial importance since the early 1900s [22]. IONPs are characterized by having a spinel crystal structure in which oxygen ions form a dense cubic packing; meanwhile, Fe^3+^ cations occupy half of the octahedral holes (hO), and M^2+^ ions are placed in eight of the tetrahedral holes (hT). In particular, copper ferrite (CuFe_2_O_4_) has an inverse spinel structure, which has a different occupation of the vacancies: M^2+^ cations are placed in hO with half of the Fe^3+^ ions, whereas the other half are in hT [23]. Among the different types of iron oxide-based NPs, the three most common structures are magnetite (Fe_3_O_4_), maghemite (γ-Fe_2_O_3_), and mixed ferrites (MFe_2_O_4_ where M = Cobalt (Co), Manganese (Mn), Nickel (Ni), or Zinc (Zn)) [24,25], where Co and Ni are toxic materials. Therefore, these magnetic materials should be functionalized with a functional chemical material.

### 2.1. Funcionalization

One of the most critical topics in designing MNPs for in vivo applications is functionalization, which provides NPs with high stability in physiological media, stealth, and vector targeting properties [26], because IONPs and SPIONs are highly reactive species with oxidizing agents such as air. Moreover, bare iron oxide has a significant toxicity effect [12], and the SPIONs have an efficiency limited by the tendency to be agglomerated. Therefore, the addition of surfactants and protective agents can serve as an appropriate surface coating to control IONPs and SPIONs stability, biocompatibility and achieve an appropriate functionalization [27,28]. In general, there are two types of structural configurations; one is magnetic core with a biocompatible polymer as coating, and the other is porous biocompatible polymer, in which the MNPs can diffuse through the pores [29].

IONPs are usually made of a crystalline core and a surface coating (e.g., dextran, citrate, chitosan, polyethylene glycol, albumin, etc.), whose dimensions are tunable to improve their stability, enhance their biocompatibility, and optimize their bio-distribution [30]. Covalent bonds can be formed between functional groups such as amine (–NH_2_), aldehyde (–CHO), carboxyl (–COOH), and sulfhydryl (–SH) on the SPIONs coating and ligands [31].

An appropriate surface coating has the potential to enable targeting capabilities of IONPs, promoting the affinity of these NPs toward a specific area [28,32]. MNPs coated with polymers have a relevant application in protein immobilization by adsorption and covalent bonding [33]. Exploring the nanoparticle shape, NPs could be helpful to other applications such as magnetic biosensor systems, magnetic particle imaging (MPI), and cell separation [34]. Furthermore, IONPs have a potential use against virus infections. It is reported as a treatment option to control influenza A/H1N1 due to their antiviral activity, determined by the change in viral RNA transcripts [35]. Using SPIONs inside of polyvinyl alcohol (PVA) nanofibers, it was found that SPIONs act as heterogeneous nucleation spots that increase the crystallization and polydispersity [36]. SPIONs were studied between rGO (reduced graphene oxide) nanosheets, showing higher stability, biocompatibility, and magneto-thermal properties for cancer treatment [37].

On the other hand, Kim et al. developed highly stable organic coated engineered SPIONs. They synthesized IONPs coated with sodium dodecyl sulfate (SDS), cetyltrimethylammonium bromide (CTAB), and polyethylene glycol (PEG), which provide adequate osmotic pressure without aggregation, reverse diffusion, or membrane blocking (by NPs) for osmotically driven membrane systems [38]. Furthermore, Piazza et al. developed superparamagnetic nanohydrogels. The maghemite nanoparticle was surface-functionalized with acrylic acid for further encapsulation with a polymeric matrix composed of derivatized dextran and acrylic acid. The magnetic nanohydrogels were obtained through vinyl polymerization with different amounts of functionalized iron oxide [39]. This material is promising for the drug delivery field.

### 2.2. Properties

Among the magnetic IONPs family, the three most popular M-IONPs are magnetite (Fe_3_O_4_), maghemite (γ-Fe_2_O_3_), and hematite (α-Fe_2_O_3_) [40]. The main characteristic of these magnetic IONPs is their strong magnetism. This phenomenon corresponds to ferrimagnetism: the magnetic moments of the different iron cations in the system are strongly coupled by antiferromagnetic interactions, but in such a way that an uncompensated magnetic moment results in each unit cell [41]. In magnetite, the Fe ions exist in the valence state +2 and +3 in a ratio of 1:2. For each Fe^2+^ and Fe^3+^ ions, a magnetic moment corresponds to four and five Bohr magnetons, respectively, for both types of ions. In addition, O^2−^ ions are magnetically neutral. The critical factor is the distribution of the spin moments of the Fe ions. The spin moments of all the Fe^3+^ ions at the octahedral positions are aligned parallel to each other [40]; however, they are directed opposite the Fe^3+^ ions of the tetrahedral positions, which are also aligned. This phenomenon occurs due to the antiparallel coupling of the magnetic moments of the adjacent Fe ions. Therefore, the spin magnetic moments of all Fe^3+^ ions cancel each other out and do not contribute to the magnetization of the solid [42].

On the other hand, all Fe^2+^ ions have their magnetic moments aligned in the same direction, and their total is responsible for the net magnetization of the material [42]. Therefore, the saturation magnetization of a ferrimagnetic solid can be calculated from the magnetic moment product of the spin of each Fe^2+^ ion and the number of Fe^2+^ ions. This would correspond to the mutual alignment of all the magnetic moments of the Fe^2+^ ions in the Fe_3_O_4_ sample [43].

However, as the size of the system is reduced to the nanoscale, each particle is considered to be a unique magnetic domain randomly oriented by the mobility of the system but ordered under the influence of an external magnetic field. Therefore, the ferrimagnetic property is lost at the nanoscale to give rise to paramagnetism [44]. Furthermore, these new unique magnetic domains have a high magnetic susceptibility (a measure of the system’s response to an induced magnetic field), thus being superparamagnetic structures.

From a chemical point of view, IONPs are very active and can readily oxidize in air, resulting in a significant loss of dispersibility and magnetism. However, magnetite is the most stable in the air for a limited time period [40]. Therefore, to avoid this drawback, the synthesis must be carried out under anaerobic conditions [45]. In addition, due to the increase in surface area to volume ratio, IONPs have a higher binding capacity and excellent dispersibility in solution. Moreover, compared with traditional iron oxides show biocompatibility and higher quality in size, toughness, monodispersity, and crystalline structure.

#### Superparamagnetims

The magnetic response to an external magnetic field depends on the prevalence and interaction between uncompensated electron spins and the system’s temperature [46]. MNPs manufactured with a ferromagnetic material, i.e., IONPs, made of magnetite (and maghemite, combining ideal biocompatibility with superparamagnetic properties [30]). Moreover, spectacular properties and superparamagnetism, such as in a high saturation field, shifted loops after field cooling, high field irreversibility, and extra anisotropy, are observed when the size of magnetic particles is in the nanodomain [47]. In other words, with nanoscale size, the MNPs are superparamagnetic with single domains, i.e., they can be magnetized under an external magnetic field and will lose the magnetization when removing the field [48]. This superparamagnetic property is related to the IONPs’ size. When the MNPs are sufficiently small (around 10–30 nm), they exhibit superparamagnetic behavior and a low magnetization per particle [49]. Superparamagnetism introduces the MNPs for promising applications such as targeted drug delivery, magnetic resonance imaging, magnetic hyperthermia, thermoablation bioseparations, and biosensing [50]. 

### 2.3. Properties Associated with Polymers

The main issue of MNPs concerning their size scales is long-term inherent instability, which occurs in two main routes: (1) dispersibility loss, where bare MNPs tend to agglomerate due to Van der Waals forces, overcoming the high surface energy and the strong magnetic attraction between particles and (2) magnetism loss, where oxidation of MNPs occurs [40,51]. These issues can be solved by the MNPs’ functionalization or by encapsulating of these MNPs into a polymeric cross-linked structure (hydrogel) known as magnetic nanocomposites (MNCs), which can also be called magnetic gels, ferrogel, or magnetoelastic gels [52]. Combining the reinforcement material and the matrix supporting the reinforcement material in the composite material, results in a better performance [3], reduces cytotoxicity, increases either the cytocompatibility and bio-conjugation [40], and improves the mechanical properties of the polymeric matrix.

The hydrogel can be chemical (covalent bonds) or physically (Van der Walls interaction) synthesized [53], adopting a three-dimensional structure, which is cross-linking through their chains. However, higher concentrations of IONPs formed more cross-links between the NPs and polymer, leading to stiffer, tougher nanocomposite hydrogels and enhanced electrical conductivity than smaller concentrations of IONPs that displays a lower cross-linked density [13]. Moreover, depending on the polymers’ nature, bonding with magnetite NPs with diameters less than 30 nm exhibits superparamagnetic behavior. The magnetization curve does not present a hysteresis curve, which means that in the absence of an external magnetic field, these particles have zero magnetization and less tendency to agglomerate.

MNCs are multi-component materials, typically containing nanosized magnetic materials to trigger the response to an external stimulus (i.e., an external static or alternating magnetic field) [54]. Furthermore, these MNCs are highly applied due to their properties. For example, thanks to their relatively large surface area and therefore high active surface sites, superparamagnetic NPs can absorb metal ions (bioremediation) to be quickly removed from a matrix using a magnetic field reused without losing the active sites [55]. Moreover, as MNCs exhibit superparamagnetic behavior, they can be used for controlled and targeted drug delivery using an external magnetic field and thus treat complex diseases [56].

## 3. Methods of Preparation

### 3.1. Synthesis of Magnetite Iron Oxide Nanoparticles

IONPs can be synthesized easily using chemical approaches such as co-precipitation, thermal decomposition, sol-gel process [57], hydrothermal [58] and polyol methods, etc. These methods require the salts of Fe^2+^ or Fe^3+^ ions or organic iron precursors and stabilizing agents that control particle size and prevent agglomeration. Unfortunately, most IONP synthesis methods involve an organic medium, making its application difficult in biological systems, whose main component is water.

The physical and chemical properties of the NPs can be controlled according to the synthesis method used to obtain the desired NPs for the desired application. In this way, monodisperse IONPs with various morphologies, including nanospheres, plates, tetrahedrons, cubes, truncated octahedrons, octahedrons, concaves, and multi branches have been successfully fabricated under different synthesis protocols [7]. In the following section, we describe in detail the most common synthetic routes of magnetite IONPs:

#### 3.1.1. Co-Precipitation

**Figure 2:**
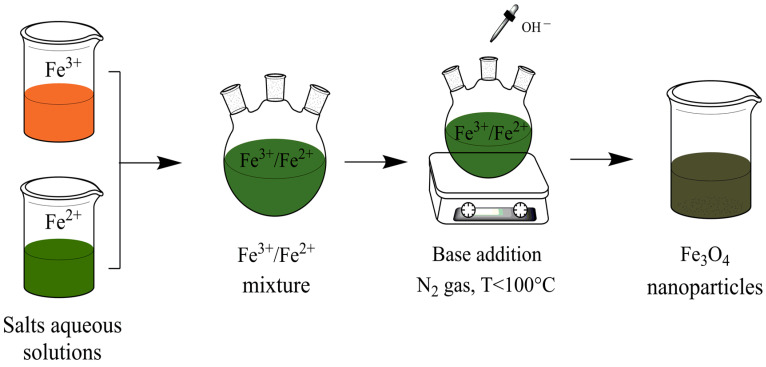
Schematic representation of MNPs. General scheme of co-precipitation method.

Due to non-toxic solvent, high yield, and easy reproducibility, this method is considered the most suitable conventional method for producing MNPs [59], for example, Fe_3_O_4_ [60]. The co-precipitation process involves ferric (Fe^3+^) precipitation and ferrous (Fe^2+^) salts aqueous solutions by adding a base. The experimental conditions include an inert atmosphere and temperatures below 100 °C. A general scheme is depicted in Figure 2. The properties of the obtained NPs, such as size, shape, crystallinity, and magnetic properties, depend on the parameters used during the reaction (pH, ionic strength, salt type, temperature, etc.) [61]. Generally, MNPs synthesized by co-precipitation will result in polydisperse particles, spherical, and have a size distribution between 5 and 40 nm [30].

SPIONPs were reported to be synthesized successfully in one study with the co-precipitation method using actinobacterial metabolites as reducing agents [62]. Rashid et al. developed IONPs using the in situ precipitation technique. First, they studied the reaction rate, where a high presence of share Fe_2_O_3_ at the start of the reaction. However, maghemite eventually is converted to magnetite by the end of the reaction, therefore enhancing the magnetic strength of the NPs [63].

#### 3.1.2. Polyol Method

The polyol method is an essential technology for producing magnetite iron oxide, where the shape and size of the magnetite can be easily controlled by changing the synthesis conditions. The experimental procedure in the polyol method is based on the dissolution of metal precursors in liquid polyols [64]. Polyols play a dual role as a reducing agent and solvent; also, they are capable of controlling particle growth [65]. Some polyols are employed to synthesize MNPs are triethylene glycol and PEG [66]. Polyalcohols have a great potential to dissolve inorganic compounds due to their high dielectric constants. They offer many advantages, such as NPs with good colloidal stability in aqueous media and polar solvents in various applications. Moreover, they present a low cost, ease of use, and, very importantly, already proven scalability for industrial applications [67]. Saddique et al. prepared spherical IONPs through the polyol method from Fe^3+^ acetylacetonate in polyvinylpyrrolidone (PVP) [68].

#### 3.1.3. Hydrothermal Method

**Figure 3:**
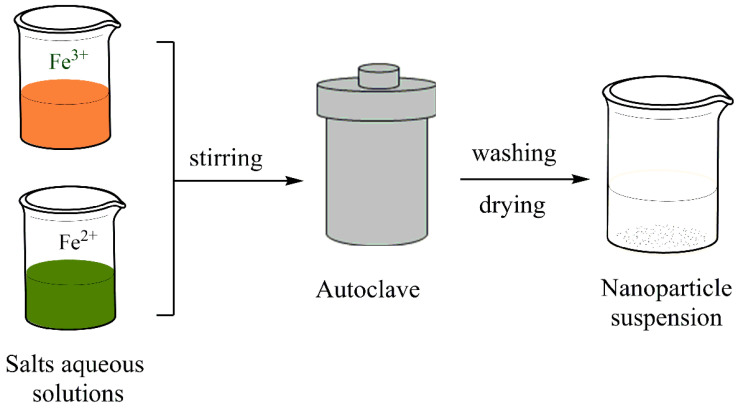
Hydrothermal synthesis of IONPs.

This versatile approach involves the synthesis of magnetite nanostructures with controlled morphologies under high-pressure/high-temperature conditions. The main advantages of this method are obtaining highly crystalline and uniform-sized NPs and the production of unstable nanocrystallites at the melting point [69]. This method corresponds to the material’s synthesis by chemical reaction performed within a sealed vessel filled with aqueous reagents under temperatures above 100 °C and autogenously high pressure [70] from 1 to 100 MPA [71]. Compared with the “low-temperature” coprecipitation procedures that usually produce poor crystalline NPs, the hydrothermal synthesis could obtain magnetic nanomaterials with very high crystallinity due to their high-temperature and high-pressure reaction conditions [72]. It is usually carried out in an autoclave or reactor, as shown in Figure 3, under conditions conducive to controlling the nucleation and growth of NPs. Through this technique, NPs are prepared with the desired shape and size by adjusting the reaction parameters (such as the precursor’s type and concentration, the precursor’s ratio to the solvent, the reaction time, and temperature) [30]. However, for the magnetic properties to be effective, the most exciting diameters are the smallest ones since the upper limit for the formation of single-domain particles is about 80 nm [73].

Furthermore, by providing high-quality iron oxide crystals, post-treatments are not required. Ozel et al. synthesized IONPs using the hydrothermal method by changing the temperature, obtaining a high crystalline morphology with a mixture of magnetite and maghemite crystalline phases. Moreover, as increases the size of the NPs, the saturation magnetization as well at low applied magnetic field [74].

#### 3.1.4. High-Temperature Decomposition of Organic Precursors

**Figure 4:**
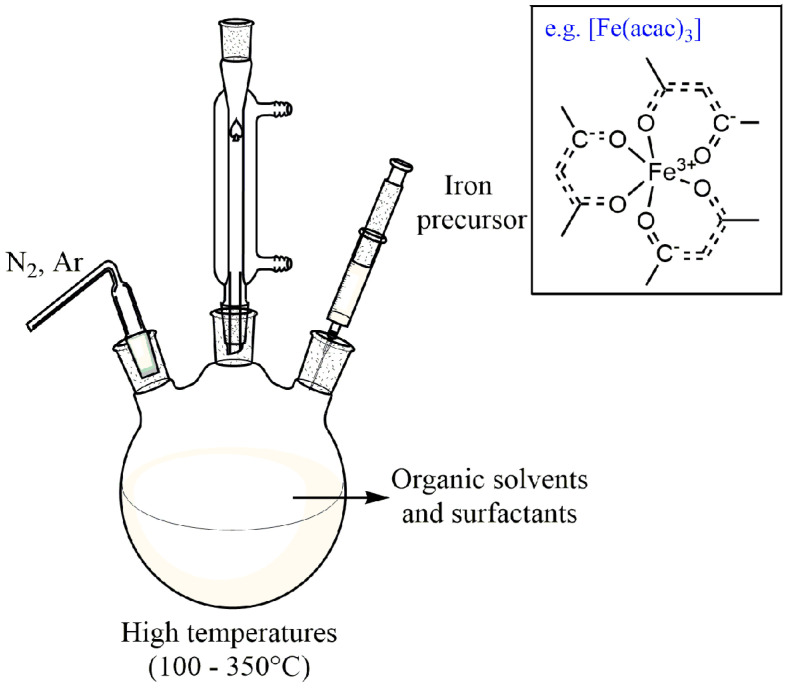
Scheme the equipment used in magnetite synthesis by high-temperature decomposition of organic pre-cursors.

Thermal decomposition is one of the precise methods for synthesizing magnetite particles with narrow size distribution and high crystal quality. However, the synthesis process requires very high temperatures and dangerous and expensive reagents [59]. In this method, magnetite NPs are formed by decomposing organometallic precursors at higher temperatures (100–350 °C) in the presence of organic solvents and surfactant capping agents [30,75], as shown in Figure 4.

This process is carried out under an inert atmosphere of nitrogen and argon (Figure 4). Usually, the precursors are Fe (cup)3, (cup = N-nitrosophenylhydroxylamine), Fe (CO)5, Fe (acac)3 (aca = acetylacetonate) (Figure 4), iron oleate, and Fe (C_5_H_5_)_2_ (ferrocene), solubilized in high boiling organic solvents in the presence of surfactants (oleic acid, fatty acids, 1-octadecene, oleylamine, and hexadecyl amine) [76]. Synthesis conditions (such as the ratio of organometallic precursors, temperature, time, and other parameters) play an essential role in the size and morphology of the particles. For example, Besenhard et al. reported the continuous thermal decomposition synthesis of IONPs using a tubular flow reactor, which provides inert reaction conditions at temperatures of up to 290 °C, and heating/cooling at rates that cannot be achieved in standard batch systems. This simple synthetic protocol was chosen using only ferric acetylacetonate, oleylamine, and 1-octadecene as a solvent and Fe(acac)3 as a precursor, but no additives to minimize costs. As a result, they obtained monodisperse IONPs of 5–7 nm [77].

#### 3.1.5. Microemulsion (ME)

Microemulsions (MEs) are isotropic and thermodynamically stable and transparent [78] dispersions consisting of two immiscible liquid phases (oil and water), are stabilized by an interfacial film of surfactant and cosurfactant molecules [79]. The presence of surfactants can form a single phase by reducing the oil-water interfacial tension. Depending on the ratio of the components, there are three types of microemulsion systems: direct (oil dispersed in water, O/W), reverse (water dispersed in water, W/O), and bicontinuous phase [79] that comprises similar amounts of water and oil. The effect of various ME parameters and components on these structures’ final size and morphology has been extensively investigated [80]. ME experiments demonstrated that the nature of the surfactant, the concentration of Fe^2+^/Fe^3+^ ions, the temperature, the pH value strongly influences the NPs size distribution and, consequently, their magnetization [81]. The main advantage of this approach is the possibility to precisely control the MNPs size [82]. However, despite the narrow size distribution that this method provides, it presents certain limitations for biomedical purposes, including requirements of low temperatures and a large quantity of oil, which limits large-scale production [16]. 

MEs are effective drug delivery vehicles since they are simple to prepare (an external energy source is not required) and are thermodynamically stable (ME phases do not easily separate over time, and a majority of microemulsions are stable for many years) [83]. The dispersed phase exists in the continuous phase in nano-droplets, which allow the encapsulation of the iron salt solution. Then, the stable nano-droplets limit the reaction medium and the nucleation and growth process of IONPs.

Lakshmanan et al. developed IONPs to remove phosphate in sewage wastewater. The MNPs display size of around 7–10 nm was synthesized using O/W microemulsion method. As a result, efficient and fast phosphate reduction was attained, while the recovery of NPs was achieved by an external magnetic field [84]. Moreover, Salvador et al. reported the synthesis of superparamagnetic magnetite NPs with average diameters between 5.4 and 7.2 nm and large monodispersity through precipitation in a W/O microemulsion, with Cetyl Trimethyl Ammonium Bromide (CTAB) as the main surfactant, 1-butanol as a cosurfactant, and with 1-hexanol as the continuous oily phase [85].

#### 3.1.6. Sol-Gel Processing

The sol-gel method is considered a feasible and inexpensive way to synthesize iron oxide from magnetite. The sol-gel method involves two main reactions: (1) hydrolysis of the precursor in the acidic or basic mediums and (2) polycondensation of the hydrolyzed products [86], followed by heat treatment. First, the hydroxylation and condensation process of the iron precursor leads to the formation of “sols” (colloidal suspensions of NPs). Then, a continuous three-dimensional lattice transformation of a sol called “gel” occurs through a drying process, and after heat, treatments are employed to obtain the nanocrystalline IONPs. Water is usually used as a solvent; alternatively, precursors can also be hydrolyzed by acids or bases [30]. The sol-gel method can be controlled by systematically monitoring the reaction parameters (such as the concentration of reactants, temperature, hydrolysis rate, and condensation reaction) [87]. This technique offers several advantages over conventional processing technologies: low reaction temperature, reasonable composition control, high purity level, and the ability to develop processes for large-area applications [88]. Calvo de la Rosa and Mercè Segarra reported an easy polymer-assisted sol-gel synthesis of copper ferrite (CuFe_2_O_4_) NPs [23]. Zhang et al. synthetized NdFeB through the sol-gel process. NdFeB gel was prepared using citric acid and glycol as the gel, and the corresponding salts of Nd, Fe, and boric acid, obtaining NPs size of 100 nm [89].

### 3.2. Fabrication of Hydrogel Magnetite Nanocomposite

The polymeric matrix known as hydrogel is prepared from the association of multiple monomer bonds to form long polymers chains that can uptake large amounts of water and other solvents [53]. Hydrophilic polymers might be considered to be those polymers that contain polar functional groups such as hydroxyl (–OH), carboxyl (–COOH), and amino (–NH2) groups that make them soluble or swelled by water [87]. Hydrogels are porous, soft, and biocompatible materials with a soft consistency similar to natural tissues. Despite their properties, low thermal stability, and poor mechanical strength constitute limitations of the hydrogel applications in biomedicine [90]

Hydrogel nanocomposites are composed of three-dimensional polymer networks, which show excellent performance due to incorporating inorganic NPs in the porous internal structure. Composite hydrogels have gained significant attention because of their enhanced intrinsic mechanical strength and bioactivity compared to pure hydrogels [91]. The composite hydrogel family includes magnetic-nanoparticle-integrated hydrogels, where these magnetic hydrogels show a magneto-responsiveness, which is observed when placed in a magnetic field (static or oscillating) [92]. Magnetic-containing polymer hydrogels have been investigated due to their great potential and exciting properties such as magnetic response, biocompatibility, mechanical properties, etc. The study reports suitable and effective methods for preparing magnetic hydrogels, including blending, in situ precipitation, and the grafting method.

#### 3.2.1. Blending Method

**Figure 5:**
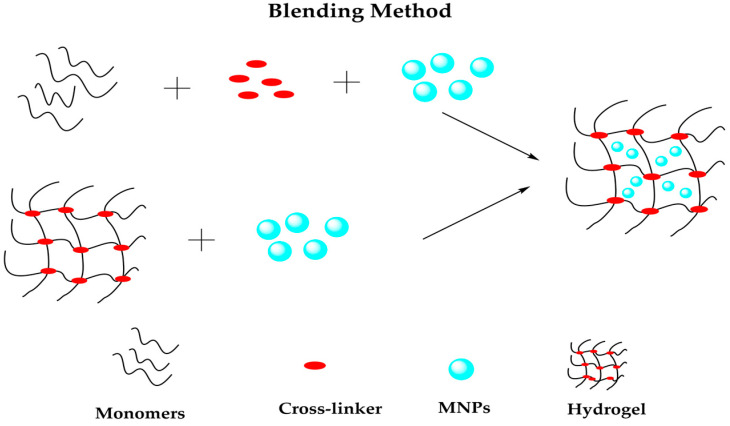
Representation of blending method to prepare magnetic hydrogels.

The blending method is a conventional route for preparing hydrogels that consists of the physical encapsulation of the MNPs into the hydrogel. This technique can be performed in two ways [93], as shown in Figure 5.

(1) Preformed MNPs to the polymer solution, causing polymer chains to cross-link and encapsulate the NPs.

(2) MNPs and network hydrogel are made separately, and after, the IONPs are trapped into the network by physical interactions.

The the incorporation of Fe_3_O_4_ NPs into a polypyrrole(PPy)/PVA polymer blend during copolymerization for As(III) adsorption from aqueous solution has been reported [94]. Moreover, solution casting was used by Shi et al. [95] to produce a hybrid hydrogel formed by bisphosphonate (BP), hyaluronic acid, and suspended Fe_3_O_4_ NPs for applications in tissue regeneration and antitumor treatment. As a result, blending is straightforward, fast, and economical compared with other tedious and time-consuming bottom-up and top-down approaches that count among the most extended methods used up to now [96]. 

#### 3.2.2. In Situ Precipitation

The method involves forming IONPs into a polymer hydrogel matrix in the presence of a precipitation medium. First, put the formed hydrogel network into a solution containing iron ions, where the molar ratio of iron ions to iron ions is 1:2 until the swelling equilibrium is reached [93]. Then, the swollen hydrogel is immersed in an alkaline solution that promotes the crystallization of magnetite. The main advantage of in situ precipitation is its simplicity and the hydrogel’s ability to load many NPs. Magnetite NPs can be obtained through (3), under the alkaline condition:

Fe^2+^ + 2Fe^3+^ + 8OH^−^ → Fe_3_O_4_ + 4H_2_O (1)

**Figure 6:**
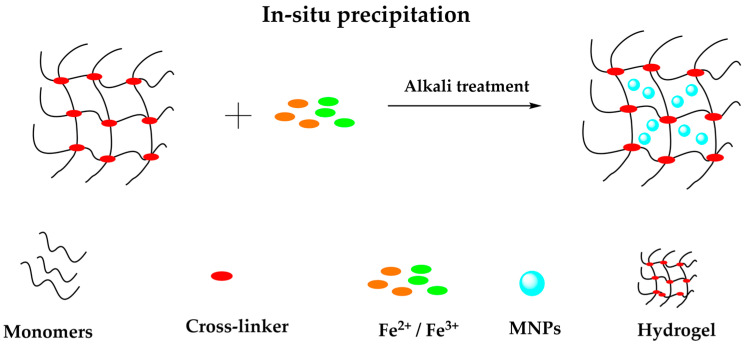
Representation of in situ precipitation method to prepare magnetic hydrogels.

In general, the excellent dispersity of MNPs in the matrix is the fundamental factor in preparing high-performance composite gels [97]. In situ incorporation of NPs has been proposed, whereby permeated ions within the hydrogel structures are reacted by drastically increasing the pH, and nanocrystals are nucleated on the functional groups of the polymer chains within the hydrogels [91], as shown in Figure 6.

Wang et al. proposed a sequential in situ route to form PPy and Fe_3_O_4_ NPs in sequence within PVA matrix for the hybrid hydrogels with decent mechanical, conductive, and magnetic properties simultaneously, known as Fe_3_O_4_/PPy/PVA hydrogel [98]. On the other hand, Freire et al. [99] elaborated a one-step process based on an ultrasound-assisted in situ method to synthesize spheroidal Fe_3_O_4_ NPs on chitosan matrix with superparamagnetic properties. Li et al. synthetized an acrylic acid/itaconic acid hydrogel. The magnetic particles were incorporated through in situ precipitation method by immersing into 0.25 M Fe^2+^ and 0.5 M Fe^3+^ aqueous ion solutions in the molar ratio of 1:2 for metal ions loading [100].

#### 3.2.3. Grafting-Onto Method

**Figure 7:**
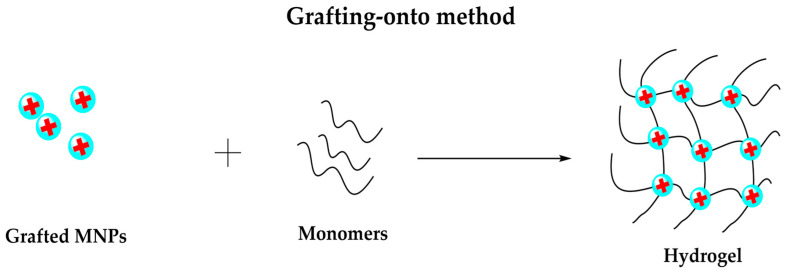
Representation of grafting-onto method to prepare magnetic hydrogels.

Unlike blending and in situ precipitation, this method involves covalent bonds between the NPs and the hydrogel matrix, as shown in Figure 7. The grafting method is based on the surface modification of MNPs with functional groups to interact with polymer chains [101] covalently. Graft refers to the reaction of a macromolecular chain with an appropriate branched or functional side group via a chemical bond [102].

The magnetic hydrogel produced by the grafting method shows higher NP dispersion stability due to the covalent coupling. However, this method involves an expensive, long, and complicated manufacturing process [103], limiting its wide application.

Tanasa et al. [104] report the synthesis of a nanocomposite hydrogel based on functionalized magnetite NPs and polyacrylamide. MNPs were prepared by co-precipitation method, grafted with 3-trimethoxysilyl propyl methacrylate for enhancing biological properties, and the hydrogel production was by free-radical polymerization. Hu et al. designed a magnetic hydrogel made from non-toxic polyacrylamide (PAAm) hydrogel and 3-(trimethoxysilyl)propyl methacrylate coated Fe_3_O_4_ via the grafting-onto approach [103,105].

## 4. Characterization

Fundamental techniques employed to investigate the IONs immersed in hydrogels include X-ray Diffraction, Fourier Transform Infrared Spectroscopy, Transmission Electron Microscopy, Scanning Electron Microscopy, Atomic Force Microscopy, Vibrating Sample Magnetometer, and Thermogravimetric Analysis, among others.

### 4.1. Structural Analysis

#### 4.1.1. Transmission Electron Microscopy (TEM)

Transmission electron microscopy involves diffraction, imaging, or spectroscopy performed with high-energy electrons in transmission geometry. It works by detecting transmitted electrons carriers of information about IONPs inner structure [30]. It is a technique employed to study NPs morphology (shape-size), dispersion, and quality [106]. Due to being an ultrasensitive technique, it can study the difference in electron density of core and shell materials. For example, in dextran-coated NPs (DINPs) micrographs, the magnetic particles can be observed as dark regions due to their high electron density compared with dextran coating [107]. The micrographs obtained can be helpful to identify adequate incorporation between the MNPs and the coatings and identify if both are biocompatible.

Furthermore, TEM allows determining the number-average diameter (Dn) and latex particles’ the polydispersity index (PDI). Simultaneously, a material’s agglomeration and aggregation state can be assessed [108]. The measurements are carried out in a dried state and in a nitrogen atmosphere. Other variations of this characterization technique were used as High Resolution-Transmission Electron Microscopy. In previous research, the morphology of IONPs was observed in hydrogels with different matrixes as chitosan [107] or chitosan-graphene [109].

#### 4.1.2. Fourier Transform Infrared Spectroscopy (FTIR)

FTIR is a technique used to characterize certain chemical groups in the hydrogel networks. The spectrum obtained through this technique shows vibration bands correlated with bonds or functional groups that are useful to identify unknown compounds. For the characterization of polymeric composites with IONPs, FTIR allows identifying the composition and functional groups involved in the capping and stabilization of the MNPs [110]. It is possible to confirm the presence of particulr groups such as amides, phenols, nitrogen, and aromatic compounds with a high binding affinity with Fe [111,112]. There are reported peaks around 410 and 683 cm^−1^ associated with the Fe-O band [113]. Fe-O vibrations at lower wavenumber bands are associated with the IONPs. It is reported that different iron oxide compounds with their respected wavenumber band. Magnetite is observed at 400 and 570 cm^−1^, maghemite band at 352, 470, and 540 cm^−1^. In the case of Fe-O, stretches for maghemite NPs are observed at 627.58 cm^−1^ [114].

#### 4.1.3. Small Angle Neutron Scattering (SANS)

Small angle neutron scattering (SANS) is a valuable technique to study magnetic and internal structural properties in nanostructures ensembles. This technique is based on the direct interaction between neutrons and the atomic nuclei, which produce a scattering length associated with specific elements [115]. It is necessary to have adequate experimental conditions such as a sample with a solvent with scattering contrast variations, which allows identifying magnetic scattering or elemental composition of individual layers [116]. SANS has been explored due to the possibility of obtaining information about intra- and interparticle magnetic moment correlations in various nanoparticle systems [117]. This technique has been performed for microscopic structures to explore polymer networks’ sizes and elucidate structures in the base of dispersion patterns. It can also complement other characterization methods such as TEM, SAXS, and DLS [118]. Among the applications of the SANS technique are the calculation of structural parameters, determination of the molecular shape, characterization of polymers, proteins, biosystems, and different states of the materials. This technique presents some similarities with XRD. However, there are considerable penetration depth and the ability to manipulate local scattering contrast by deuterium labeling without significantly affecting the chemical interactions, resolution, and length scales suitable for polymer studies. Selective Deuterium is used when there is a weak scattering of the components of interest [115]. SANS analyzes interparticle interactions as a function of the nanoparticle volume fraction.

#### 4.1.4. X-ray Diffraction (XRD)

X-ray diffraction is widely used to characterize polymorphic structures, identify phases, and purity of the sample. The XRD pattern for NPs was observed under a wide range of Bragg’s angle (θ) [106]. Orientation is a critical factor determining material’s mechanical properties, dimensional stability, and diffusion behavior.

XRD helps to identify the physical and chemical form of the NPs as iron oxides, hematite, or maghemite structures or to identify the mechanism for metal binding on NPs [112]. X-ray Diffraction allows determining crystallite size from the full width at half maximum (FWHM) using the Scherrer Equation (2).

(2) $$D=\frac{K\lambda }{\beta \cos\theta }$$

D corresponds to the average crystallite size, K = 0.9 is a constant related to the crystallite shape, λ corresponds to X-ray wavelength, θ is the Bragg’s angle, and β is the peak width of the diffraction peak profile at half maximum height [119]. A dispersion of IONPs in chitosan solution was reported, which present six characteristic peaks identified by their crystal planes (220), (311), (400), (422), (511), and (440) which correspond to iron oxide [120].

#### 4.1.5. Scanning Electron Microscopy (SEM)

This technique studies the interaction of electrons and the surface of the material. The micrographs obtained results from a series of elastic and inelastic signals, which give us morphological, compositional, and structural information [121]. The micrographs obtained from secondary electrons are related to the topographic information of the sample surface. This microanalysis technique analyzes the morphology of magnetic particles dispersed in the matrix and the surface texture of hydrogels [122]. The conventional characterization process requires vacuum conditions and the use of samples subjected to drying at high or low temperature or critical temperature at which their porous structure is preserved [123]. SEM images identify morphological information such as shape or size or IONPs and identify phenomena such as agglomeration, which can be associated with electrostatic interaction between layers of NPs [124].

#### 4.1.6. Thermogravimetric Analysis (TGA)

Thermal characterization provides information about the thermostability and the average mas content of specific components. Thermograms obtained are helpful to determine the stages of thermal degradation at determined temperatures [120]. There is a first degradation related to pendant groups (amide and hydroxyl ethyl groups) and a second degradation associated with the main chain [122].

#### 4.1.7. Fluorescence Microscopy

Fluorescence is a luminescence process through which it is possible to identify specific components from complex materials such as hydrogels. The technique is founded on source light to excite an electron. Electrons are promoted to an excited state, and the molecule will be in an excited vibrational energy state. A photon will be emitted when the molecule comes back to its ground electronic state [125].

Confocal fluorescence microscopy provides three-dimensional images of objects stained with fluorophores. Usually, the maximum observable depth with this technique is close to 200 µm, and it is improved with two-photon fluoresce microscopy, which enhances penetration depth. Additionally, it gives us information about wall thickness and macropore size, which is helpful to relate to swelling-deswelling mechanisms. Fluorescence labeling has been a handy tool for visualizing cellular structures and processes. In addition, fluorescence groups can be linked to magnetite-polymer hybrid NPs with the main application in drug carriers.

### 4.2. Magnetometric Methods

#### Magnetic Response Measurements

Materials generally show magnetism only in the presence of an applied field. However, certain materials exhibit ordered magnetic states in the absence of an applied field. Those materials that present magnetization without a magnetic field are ferro or ferrimagnets. Based on the magnetic susceptibility, there is a general classification: paramagnetic and diamagnetic.

The most common characterization techniques to measure magnetic parameters of IONPs are Vibrating Sample Magnetometer (VSM) and Superconducting Quantum Interference Device (SQUID). Saturation magnetization, remnant magnetization, and the coercive field are some magnetic parameters that can be deduced from hysteresis loops. Moreover, it helps identify the type of magnetism presented, such as diamagnetism, paramagnetism, ferromagnetism, antiferromagnetism, and ferrimagnetism [30]. MNPs are known for their superparamagnetic properties. When MNPs apply magnetic field, they show colloidal behavior but uniform dispersion when the applied field is retired [126].

### 4.3. Swelling Analysis

The swelling properties are used to define the characteristics of hydrogels. Some factors related are network density and polymer-solvent interaction parameters. Hydrogels are soft and wet polymeric materials, showing a finite swelling capacity. In the 1940s, the research of 1094 Nobel laureate Paul Flory led to a detailed, fundamental understanding of the hydrogels’ cross-linked structure, their swelling/syneresis characteristics, and the small and large deformation behavior in pure water and physiological fluids [127]. The ability of gels to retain fluids could be analyzed through swelling behavior characterization. The swelling capacity of hydrogels varies considerably depending on different factors. It is reported that the swelling capacity of the hydrogel in water decrease when the gel content of Carboxymethylcellulose (CMC) hydrogel increases, which results in the increment of the cross-linking degree of CMC [128]. The lower swelling limit is related to the dried state, and the upper swelling limit depends on its network and contour length [129]. Swelling and mechanical properties are characterized based on the swelling ratio (Sr) (3), defined as the fractional increase in the weight of the hydrogel due to water absorption.

Sr = (W_s_ − W_d_)/W_d_ (3)

where W_s_ corresponds to the weight of the swollen hydrogel, while W_d_ corresponds to the weight of the dry hydrogel, the swelling ratio relates to tensile properties, specifically with network elasticity [129]. Swelling capacity increases with polar functional groups, such as hydroxyl, carbonyl, and epoxy, which give a highly hydrophilic nature [109]. The temperature has a relevant effect on swelling percentage. There is a variation in the swelling percentage when hydrogels are loaded with magnetite particles [122].

### 4.4. Cytotoxicity Analysis

Many synthetic methods to produce the MNPs could produce cytotoxic effects caused by free-radical production and high iron dosage. Previous studies reported that a dose level of iron up to 100 µg/mL results in nontoxic effects in vivo and in vitro conditions. Biocompatible composites could be produced by ligand exchange or encapsulation methods, where water-dispersible IONPs are obtained [130]. Polymeric hydrogels have numerous potential applications, but they require a previous cytotoxic analysis. Their characterization can be carried out directly or indirectly. The direct form involves direct contact tests with the cells, such as human epithelial cells, and the indirect ones are carried out with the growth of cells in polystyrene plates [131]. Another critical test is related to the toxicity of the NPs immersed in the material. Finally, an overall process is carried out by fluorescence microscopy, where dead cells and dead cells are recognized, with which a cell viability index can be deduced.

## 5. Applications

There is an interest in IONPs with polymers obtained from natural sources due to their higher biocompatibility which implies the potential use on biomedicine. NPs are commonly used in different biomedical applications because of their anticancer, antimicrobial, antiviral, antiplasmodial properties. Moreover, magnetic iron oxide is already approved by the food and drug administration (FDA) for medical and food applications, making IONPs good candidates to study their biofilm inhibitory properties [132].

MNPs have been used in biomedicine since the 1990s. The fact that iron is easily metabolized within the body that the particles have sizes comparable to that of proteins, cells, viruses and DNA that the surface of these particles can be modified in order to bind molecules of biological interest that particles possess a high magnetic moment, as well as the fact that the field lines can cross the human body, means that the particles hold a promising future in the search for minimally invasive methodologies to assist in the diagnosis and treatment of diseases.

Recently, applications have appeared that combine therapeutics and diagnosis, allowing a high control of the effectiveness of individual treatment. This combination is now known as theranostic. In the absence of a coating, the MNPs show hydrophobic surfaces that facilitate the attractive Van der Waals force and give rise to the formation of agglomerates that can reach micrometric values. This aggregation occurs especially in biological fluids due to salts and plasma proteins. Under these circumstances, MNPs are incompatible with their use in biomedical applications due to the high possibility of clogging tiny capillaries.

Biomedical applications of MNPs can be classified according to whether they are applied inside or outside the body (in vivo or in vitro). The primary use in in vivo applications is selection and separation in diagnostic and magnetorelaxometry applications, while in vivo applications can be separated into therapeutic and diagnostic applications [16,30,133,134]. Magnetic hyperthermia, magnetic resonance imaging, and drug delivery could be the principal biomedical applications studied.

### 5.1. In Vivo Applications

MNPs must be compatible and easily biodegraded in the body for therapeutic applications. In IONPs, after being metabolized, iron ions are added from the body’s iron stores and are finally incorporated by erythrocytes as part of hemoglobin. Of these particles, hardly any harmful effects have been described. The cytotoxic effects observed due to the ingestion of this type of particles only occur at high concentrations (greater than 100 ug/mL) [135]. Two types of superparamagnetic IONPs are used: SPIONs and USPIONs (Ultra SuperParamagnetic IONPs), differences only in size (USPIONs, <50 nm; SPIONs, >50 nm).

Size plays a crucial role in biodistribution in vivo since the residence time in the organism depends on the size of the particle. According to the therapeutic purpose of the administration of the MNPs, a parameter to be taken into consideration for them to be of interest from the clinical point of view is that the circulation time in the blood after being injected into the body is long enough to that they can achieve their desired goals [136]. Otherwise, its therapeutic efficacy would be reduced. When a particle enters the body, it is recognized by a set of proteins called opsonins. After binding, the proteins serve as a decoy for the action of the cells of the macrophage-phagocytic system that cause the internalization via endocytosis of the MNPs; these are grouped in the lysosomes where presumably, they are degraded, at low pH, to iron ions by a series of hydrolytic enzymes by the endogenous pathways of iron metabolism. This system includes macrophages from the liver, spleen, and lymph nodes and is responsible for recognizing and eliminating all foreign particles that enter the body and, therefore, also causes the elimination of MNPs. Thus, one way to increase the bioavailability of MNPs is to prevent them from being eliminated by the macrophage-phagocytic system [137].

The size of the MNPs must be small enough to avoid capture by the spleen (less than 200 nm) but large enough to avoid direct filtration by the kidneys (greater than 5 nm). Therefore, MNPs ranging between 10 and 100 nm have the most suitable size to achieve optimal distribution in vivo.

Another way to prolong life in the body is to coat NPs with biocompatible polymers or encapsulate them in liposomes. The polymeric shell or the encapsulation in liposomes provides a physical barrier that prevents the agglomeration of the NPs and enables their easy dispersion in physiological solutions. Biodegradable polymer-coated MNPs show lower toxicity and have higher biocompatibility than uncoated inorganic NPs [138]. The polymers most commonly used as coatings for NPs are dextran, used for its high biocompatibility and high affinity for iron, and PEG, with a high non-stick capacity that reduces the uptake of particles by macrophages, increasing their circulation time in the blood. Other polymers commonly used as coatings for MNPs are PVA and chitosan, which provide a biocompatible, cationic and hydrophilic shell.

#### 5.1.1. Therapeutic Applications: Hyperthermia/Ablation

Magnetic hyperthermia is the phenomenon that occurs when an area containing MNPs is exposed to electromagnetic waves with a frequency equal to several hundred MHz, an interaction that generates heat in the area by dissipating energy and allows temperatures between 42 and 45 °C to be reached for a minimum period of 30 min. IONPs are used to induce local heat enhancement when submitted to another magnetic field. It is efficient to eliminate cancer cells that cannot survive in a certain temperature range. Good control of the temperature both inside and outside the tissue to be treated is of the utmost importance since the aim is to produce the increase only in a delimited area. Tumor cells are more sensitive to temperature increases than healthy cells; thus, the main advantage of magnetic hyperthermia is that it allows heating to be restricted to the tumor area [139]. The use of IONPs with polymer (i.e., Polyethylenimine (PEI), Chitosan, and Polyphenol) coating increments the efficiency of photothermal ablation [140]. Hyperthermia is used to enhance established therapies such as radio or chemotherapy. Tumor cells of a hypoxic nature (with low oxygen levels) are resistant to radiation; however, heat destroys hypoxic cells and normal cells alike. It has been shown that a lower dose of radiation is required to destroy the same proportion of tumor cells when previously subjected to hyperthermia processes [141].

Another of the essential requirements that must be considered is that the temperature in the zone of attention does not rise too much, since if a higher temperature than the established temperature is reached, the phenomenon called thermal ablation will occur, which occurs when the temperature reached is around 50 °C. In this case, in addition to cell death, necrosis occurs. Thermal ablation has been used as salvage therapy after post-radiotherapy failure. Hyperthermia, in addition to the application of electromagnetic fields, can be produced by ultrasound, perfusion therapy, interstitial laser photocoagulation, and external contact heat administration [142,143]

MNPs thermotherapy is a new, minimally invasive option. The generation of heat depends on the magnetization properties of the specific formulations of the NPs and the intensity and frequency of the magnetic field. The use of nano-sized (single-domain) particles is preferred over micro-sized (multi-domain) materials, as NPs absorb much more power at tolerable AC magnetic fields. It is of paramount importance to make use of well-established synthetic routes that produce uniform particles in order to be able to have rigorous temperature control.

#### 5.1.2. Drug Release

IONPs have many essential advantages due to their magnetic properties. Nevertheless, the challenge in using this nanoparticle as a therapeutic agent for a drug delivery system to the specific tissue or cells is due to the bare iron oxide’s poor stealthiness and the significant toxicity effect [20]. One of the most critical topics in designing IONPs for in vivo applications is functionalization, which provides NPs with high stability in physiological media, stealth, and vector targeting properties [26]. The polymers most commonly studied for drug delivery are from natural proteins or polysaccharides are chitosan, alginate, or synthetic polymers such as PLA, PGAR, or PLGA [144]. The possibility of using external magnetic attraction or the functionalization of MNPs with molecules that recognize targets on which to act makes it possible to guide the NPs toward the areas of interest where drug release should occur. This function of focused release of therapeutic drugs leads to a reduction in the dose of the drug and the disappearance of unwanted side effects on other healthy cells or tissues [145].

Guidance is related to the movement and trajectory that NPs can experience when applying a static or alternating magnetic field so that they acquire a speed and can be manipulated by controlling said field. The same effect can be achieved if the particles are targeted, for example, if they present on their surface a specific antibody for an antigen present on the cells or tissue to be treated. As a consequence of the guidance of the MNPs, they can be focused, i.e., concentrated in a specific place in the organism [146].

As previously mentioned, the surfaces of MNPs can be modified with organic polymers to make them biocompatible and suitable for subsequent functionalization by linking bioactive molecules. The drug localization process using magnetic delivery systems is based on the competition between the forces exerted on the particles by the blood behavior and the magnetic forces generated by the magnet [147]. Polymeric-coated MNPs have different pathways for cellular internalization such as endocytic, pinocytic, phagocytic, or receptor-mediated; it will depend on the size and surface charges [148].

#### 5.1.3. Diagnostic Applications: Magnetic Resonance Imaging

Magnetic Resonance Imaging (MRI) is one of the most widely used techniques today as a non-invasive diagnostic tool. IONPs are useful as a contrast agent. It is based on the difference in nuclear magnetic relaxations of protons in water between biological fluids and solid tissues. A contrast agent modifies the rate of nuclear magnetic relaxation of the protons in its environment and changes the contrast of the signal. MNPs are administered to increase the image contrast between healthy and diseased tissue and/or indicate the functional status of organs or blood flow [149].

MNPs as contrast agents for MRI allow a better interpretation of the images obtained by increasing the differences between normal and pathological tissues [150]. Two types of contrast agents can be distinguished: T1 contrast agents (paramagnetic metal ions, such as Gd^3+^) and T2 contrast agents (MNPs). The T1 contrasts increase the image’s brightness, while the T2 contrasts give negative images. Conventional MRI contrast agents are most effective in a single imaging mode, T1 or T2. However, the combination of the simultaneous use of both agents in a single contrast agent can represent a significant innovation since it can potentially provide more precise images. The foundation would be based on the strong magnetic coupling between the contrast agents T1 and T2 when close. Then, the spin-lattice relaxation processes of the T1-type materials cause the decrease of this contrast [151].

On the other hand, MNPs tend to accumulate in places where there is an alteration of the tissue’s vasculature, inflammation sites, tumor lesions, and lymph nodes [126]. This phenomenon, together with the fact that there is a defective lymphatic drainage system in the tumor tissue, causes the accumulation in the tumor areas of those MNPs with sizes between 10 and 100 nm [149,152].

### 5.2. In Vitro Applications

#### Separation and Selection

Solid-phase extraction (SPE) is highly popular as an efficient system for isolating and pre-concentrating the desired components of an analytical sample, providing an excellent alternative to conventional concentration methods such as liquid-liquid extraction. For example, SPE is a routine extraction method for determining trace level contaminants in environmental samples. However, the separation and preconcentration of a substance present in large volumes of the solution are time-consuming when using a standard SPE column. This is where the use of magnetic or magnetizable adsorbents, formed by MNPs, becomes essential, giving rise to the so-called magnetic solid-phase extraction. In this procedure, the magnetic adsorbent is added to the solution or suspension that contains the substance to be separated. This is absorbed on the magnetic adsorbent, and then the adsorbent with the adsorbed substance is recovered from the liquid phase using an appropriate magnetic separator. For separation and selection, the advantage of using MNPs instead of magnetic microparticles lies in being able to prepare suspensions that are stable against sedimentation in the absence of an external magnetic field [153,154].

## 6. Perspectives and Future Challenges

This work presented the main techniques for preparing and characterizing IONPs and their corresponding nanocomposite based on cutting-edge research articles and reviews. The study of IONPs with a specific high magnetic moment, specific surface properties, and biocompatibility represents a technical challenge. The use of IONPs presents different limitations as low stabilization, tendency to form agglomerations, toxicity, and the loss of magnetization. The microscopic magnetic behavior of NPs could differ from the macroscopic properties compared with theoretical bulk values [155]. The properties of MNPs will depend on size due to the relative proportion of superficial atoms concerning their volume [156]. The use of organic and inorganic molecules such as polymers, surfactants, or biomolecules has been studied as protecting agents from stabilizing the nanomaterial, avoiding aggregation, and tolerating changes in the pH and electrolytes presence be able to obtain an adequate chemistry surface [157]. The polymer could be one of the best options by the chemical and thermal stability of the NPs, because it reduces the tendency of aggregation and increases the possibilities of use [33]. However, the use of polymeric coating as protecting agents results in the increment of NPs size and, consequently, a lower magnetic property. Therefore, the polymer can adopt a cross-linked structure (hydrogel) as a supporting matrix for encapsulation the MNPs. These magnetic hydrogels have a magnetic-sensitive smart responsiveness which is suitable for huge applications [158].

The massive production of MNPs has been restricted by slow procedures, high costs, and low yield. Therefore, it is necessary to study known synthetic processes or alternative routes to control nanoparticle characteristics such as composition, size, shape, magnetization, and surface charge [119]. Most of the chemical and physical methods used to produce nanomaterials are toxic and involve high-energy consumption; for that reason, the major challenge consists of developing eco-friendly methods. These methods must be close to green chemistry [159], i.e., to adapt the methods to the use of water as a sustainable and suitable medium for producing these materials. Another challenge presented by this technology is production on an industrial scale. The gap between laboratory-scale (scientific production) and industrial-scale (“real life”), and its subsequent application, must gradually disappear. Therefore, it is necessary to perform computer simulations, cost analyses, viability, feasibility, and determine target groups that would benefit from implementing and developing these materials. Within this field, optimizing resources will be a critical factor in the large-scale production process. Studies reported are principally focused on NPs with spherical morphology, but there is an interest in anisometric NPs, which could potentially apply to the biomedical area [34]. It seems that MRI could be the most critical application for imaging techniques such as computed and positron emission tomography due to being a potential contrast agent with low toxicity compared with gadolinium which is commonly used [160].

## 7. Conclusions

IONPs and SPIONs are MNPs with highly magnetic properties. These NPs can be obtained by co-precipitation, polyol, hydrothermal, microemulsion, and sol-gel methods, which require iron ions as precursors and stabilizing agents. These MNPs display different physicochemical properties, which can be controlled by adjusting the synthesis parameters such as temperature, molar ratio, precursors, reaction time, etc. The most conventional method to prepare MNPs is co-precipitation due to ease of manufacture and reproducibility. Despite their potential features, these MNPs present some disadvantages, such as toxicity not being stable in aqueous media, resulting in easy aggregation and precipitation. Therefore, to overcome this problem, MNPs must be coated or encapsulated with surfactants, protective agents, or cross-linking matrixes to improve their properties. A synergistic approach between MNPs and hydrogels would enhance the performance of both materials.

Hydrogels are known for their smart response when subjected to an external change. In this case, when hydrogels are combined with MNPs, they form a magnetic composite that adopts a magnetic behavior, showing a stimuli-response when subjected to a magnetic field. The synthesis of these composites is based mainly on blending, in situ precipitation, and grafting methods, where the blending method is the most common to produce magnetic hydrogels. Blending and in situ precipitation are low cost and straightforward methods for synthesizing magnetic composites. In contrast, the grafting-onto method requires an expensive and struggling manufacturing process. In addition, some characterization techniques are necessary to understand and study the functional groups’ surface morphology, chemical composition, and spatial distribution.

These MNPs are highly used in in vivo and in vitro applications in biomedicine. They show a better diagnosis performance, mainly as MIR, because MNPs are used as contrast agents. Moreover, they are highly used in drug delivery. When these NPs are functionalized with polymers, they can interact with bioactive molecules that recognize targets and act on specific sites.

## Data Availability

Not applicable.
